# Preliminary clinical research on epiphyseal distraction in osteosarcoma in children

**DOI:** 10.1186/1477-7819-12-251

**Published:** 2014-08-07

**Authors:** Songtao Gao, Yan Zheng, Qiqing Cai, Weitao Yao, Jiaqiang Wang

**Affiliations:** 1 Department of Orthopaedics, The Affiliated Tumor Hospital of Zhengzhou University, Zhengzhou, Henan 450008, China; 2 Department of Radiology, The First Affiliated Hospital of Zhengzhou University, Zhengzhou, Henan 450052, China

**Keywords:** Epiphyseal plate, Limb length, Joint, Osteosarcoma

## Abstract

**Background:**

The feasibility of distal femur epiphysis preservation through epiphyseal distraction by external fixator in childhood osteosarcoma was explored.

**Methods:**

Between July 2007 and May 2011, 10 children who were suffering from distal femur osteosarcoma received epiphyseal distraction by external fixator, combined with tumor resection and repair with massive allograft bone to preserve the epiphysis of the distal femur and knee function. There were six male and four female patients, 9- to 14-years old (average 10.5 years old). The tumors were staged clinically according to the Enneking staging method: six cases were classified as stage in IIA and four cases as stage in IIB. All patients were diagnosed by biopsy, then received chemotherapy before and after surgery. All patients received tumor bone resection and the defects of the bone were repaired with massive allograft bone that was fixed by intramedullary nails; the distracted epiphysis and allograft bone were fixed with cancellous screws.

**Results:**

All cases received follow-up from 15 to 56 months (average 38.5 months). There were no local recurrences. One case died of lung metastasis and one case had poor incision healing for rejection of allograft bone. According to the functional evaluation criteria of the International Society of Limb Salvage (ISOLS) after operation, five cases were rated excellent, four cases good and one case fair. The ratio of excellent or good was 90.0%. There was no statistically significant difference in length between the operated and the normal lower limbs during the last review.

**Conclusions:**

Epiphyseal distraction by external fixator can result in satisfactory limb length and joint function for children with a malignant bone tumor.

## Background

Metaphysis is the predominant site of malignant bone tumors in children. In the past, the epiphysis had to be resected to obtain a complete tumor excision with clear margins when the adjacent metaphysis was involved in a malignant tumor. This inevitably resulted in a discrepancy of limb length or dysfunction of the involved joints [1-3]. At present, whether the epiphysis of the tumor-bearing bone can be preserved so that the function and growth capability of the involved joint can be preserved has been a new challenge to the clinician [4]. This research was dedicated to epiphyseal distraction by an external fixator in children with a distal femur osteosarcoma to preserve the epiphysis of the distal femur and knee joint function. Factors such as tumor control, postoperative complications, knee joint function and limb length were analyzed as well.

## Methods

### General patient data

From July 2007 to May 2011, we assessed 10 distal femur osteosarcomas of children who were treated using epiphyseal distraction by an external fixator, combined with tumor resection and repair with a massive allograft bone to preserve the epiphysis. Mean age of the patients (six boys and four girls) was 10.5 years old (range 9 to 14 years). All patients in the studies had been approved by the ethics committee of the affiliated tumor hospital of Zhengzhou University and, therefore, the studies were in accord with the ethical standards laid down in the 1964 Declaration of Helsinki and its later amendments. All patients gave their informed consent prior to their inclusion in the study. The study was approved by the affiliated tumor hospital of Zhengzhou University institutional review board and the informed written consent was obtained from all legal guardians. All patients were diagnosed by biopsy before and after surgery. X-ray, computed tomography (CT) and magnetic resonance imaging (MRI) were accomplished preoperatively to determine the extent and clinical grading of the tumor. whole-body bone scanning by Emission Computed Tomography (ECT) and chest CT were performed to determine whether there was a metastasis. All tumors were staged according to the Enneking method: IIA for six cases, IIB for four cases. The extent of tumor invasion was also classified according to the image staging method of San-Julian [5] and determined to be type I for five cases and type II for five cases. All patients received neoadjuvant chemotherapy according to the COSS 86 scheme [6]. The effects of chemotherapy were evaluated by the Rosen method [7].

### Inclusion criteria of patients

**Figure 1:**
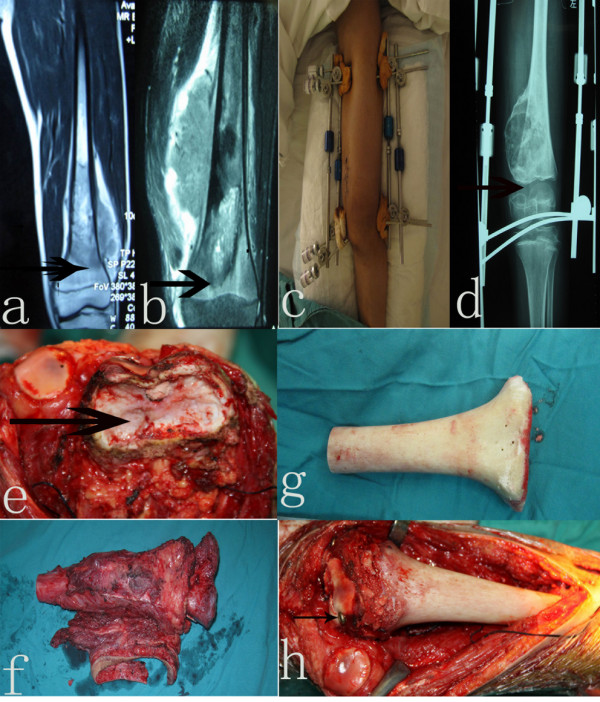
**Epiphysis distraction before surgery and fixation between the epiphysis and allograft bone during surgery. a)** Type I of distal femur osteosarcoma (arrow point shows the gap between osteosarcoma and the epiphyseal plate >2 cm). **b)** Type II of distal femur osteosarcoma (arrow point shows the gap between osteosarcoma and epiphyseal plate <2 cm). **c)** Gross view of epiphysis distraction by an external fixator. **d)** X-ray of epiphyseal distraction by an external fixator(arrow point shows the metaphyseal and epiphyseal plate were completely separated). **e)** Appearance of proximal epiphysis section (arrow point indicates a cross section of the proximal end of the epiphysis was irregular sawtooth shaped). **f)** Appearance of tumor bone after resection (the interval between osteotomy level and tumor border was no less than 5 cm). **g)** The distal part of the allograft bone was trimmed to be consistent with the proximal epiphysis. **h)** Appearance after fixation between the epiphysis and allograft bone (arrow point shows the fixation of cartilage and allograft bone by lag-screw of cancellous bone).

The patients had to meet the following criteria: 1) the physis of the involved distal femur was open; 2) tumors were sensitive to preoperative chemotherapy; 3) the location of the lesion was classified as typeI or typeII of image staging using the San-Julian method [5] (Figure 1a,b); 4) after two weeks of preoperative neoadjuvant chemotherapy, laboratory results, such as routine blood, hepatic and renal function, serum electrolytes, blood coagulation function and accessory examinations, such as electrocardiograms, of the patients were normal; and 5) there was no local (at the biopsy position) or general infection in the patients.

We depend on the preoperative MRI after neoadjuvant chemotherapy [8] to determine the interval between the tumor and distal femur epiphysis. Pathological examination during operation was used to determine whether there was a margin of the surgical area. If the margin is positive, the preservation of the distal femur epiphysis should be abandoned and the epiphysis should be resected at once. All surgical margins in this research were negative during the intra-operative and postoperative pathological evaluation.

### Operation method

#### Epiphyseal distraction by external fixator

The distraction was performed according to the Cañadell method [9] under C-armed monitor. After preoperative chemotherapy, two Steinman pins of 4 mm diameter were drilled in parallel through the anterior and posterior 1/3 part of the sagittal section of the distal femur epiphysis. Another two pins were drilled in parallel through the proximal femur shaft 8 cm away from the proximal border of the tumor. The interval between pins on the same side was 1 cm. All pins were fixed by four longitudinal rods and screw nuts (Figure 1c). The rods were elongated at a rate of 2 mm two times a day until a complete distraction between epiphysis and metaphysis was obtained. In general, the distraction was obtained in five to seven days and the allogenic bone was prepared during this period of time.

#### Evaluation criteria of epiphyseal distraction

The criteria included: A sustainable feeling of pain and a tearing sensation in the operated knees; the gap between the epiphysis and metaphysis of the distal femur was increased as can be perceived by palpation; the gap was no less than 2 cm and the distraction was confirmed by X-ray [9] (Figure 1d).

#### Tumor resection and reconstruction

After the tumor was resected, the bone defect was reconstructed with massive allograft bone and fixed by locking intramedullary nail. First, the Steinman pins and external fixator were removed after local sterilization, the lesion was exposed and the gap between the epiphysis and metaphysis of the distal femur can be observed: both ends of the proximal epiphysis and distal metaphysis were irregular sawtooth shaped (Figure 1e). The epiphysis was isolated and osteotomy of the tumorous bone was accomplished according to the scope determined preoperatively (the interval between the osteotomy level and the proximal tumor border was no less than 5 cm) (Figure 1f). A long segment of sterilized allograft bone was used to repair the bone defect. The distal part of the allograft bone was trimmed to be consistent with the shape of the proximal end of the epiphysis (Figure 1g). All the allograft bone was fixed by a locking intramedullary nail, after two cartilage flaps about 5 mm diameter were incised at the internal and external surface of epiphyseal cartilage, respectively. The epiphyseal cartilage and allograft bone were fixed vertically by four cancellous bone screws of 4 mm diameter and the ends of the screws were covered by the restored cartilage flap (Figure 1h). Autogenous iliac bones were transplanted to two ends of the allograft bone to accelerate the healing of the bone.

#### Postoperative treatment

**Figure 2:**
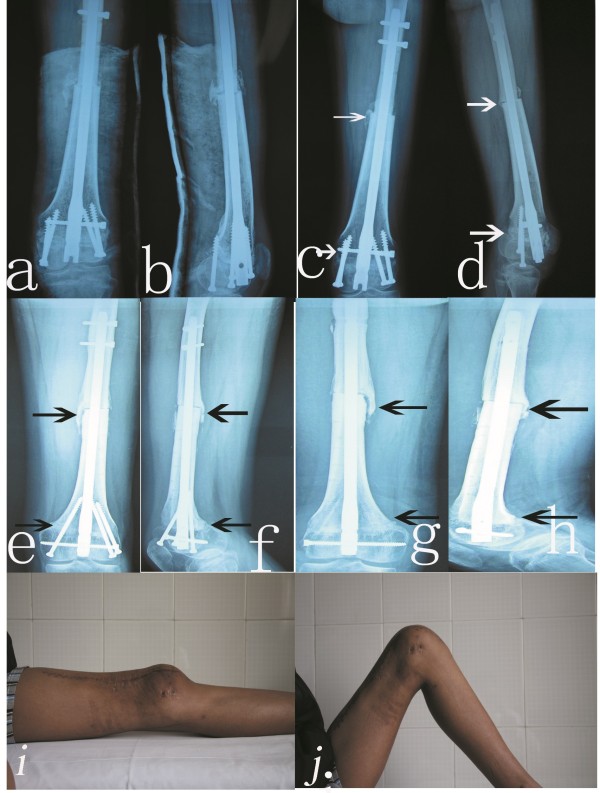
**Healing of the bone and motion of the knee joint after surgery. a,b)** The X-ray appearance two weeks after operation (the operated limb was externally fixed in the functional position by plaster). **c,d)** The X-ray appearance six months after the operation (arrow point shows complete healing of the gap between the epiphysis and allograft bone; there was considerable callus formation in the gap between the allograft bone and the proximal femur). **e,f)** The X-ray appearance one year after the operation (arrow point shows complete healing of the epiphysis and the allograft bone; there was considerable callus formation in the gap between the allograft bone and the proximal femur). **g,h)** The X-ray appearance two years after the operation (arrow point shows complete healing of the epiphysis and allograft bone; there was considerable callus formation in the gap between the allograft bone and the proximal femur after the proximal locking screw had been removed). **i,j)** The range of motion of the knee joint three years after operation.

The operated limb was held in the functional position by plaster for six weeks (Figure 2a,b), functional training of the quadriceps femoris was carried out in the early period after surgery, and knee joint exercises were performed after the plaster was removed. Partial weight bearing of the operated limb under the protection of a brace was encouraged at eight weeks after the operation and full weight bearing of the operated limb was carried out only after bone healing had been confirmed by X-ray.

### Patient outcome

#### Tumor control

Whether there was local recurrence or distant metastasis of the tumor after operation was evaluated.

#### Complications and bone healing

The healing of the incision and the bone as well as complications, such as infection, looseness or rupture of internal fixation and fracture of the bone, were evaluated.

#### Limb function and joint stability

The functional score of the operated knee was assessed according to ISOLS criteria [10].

#### Limb length

The length of the operated limbs was compared with that of the contralateral limbs at every examination after surgery. The limb length at the last examination was analyzed using SPSS12.0 statistical software. T-test was adopted in group measurement data, *P* <0.05 was considered statistically significant.

## Results

### Tumor control

All 10 patients were followed up for 15 to approximately 56 months, average 38.5 months. There was no local recurrence for any patient; one patient died of lung metastasis two years after surgery.

### Complications and bone healing

One patient suffered from local infection and exudation from the wound, but the status improved after anti-inflammation treatment. There was no looseness or rupture of internal fixation or fracture of the bone. X-ray imaging showed complete healing between the epiphysis and allograft bone at six months after surgery. There was callus between the allograft bone and proximal femur (Figure 2c,d). There was complete healing of the epiphysis and allograft bone and considerable callus between the allograft bone and proximal femur one year after the operation (Figure 2e,f). There was complete healing of the epiphysis and allograft bone after the vertical fixed screw was removed two years after surgery, and there was considerable callus between the allograft bone and proximal femur after the proximal locking screw had been removed (Figure 2g,h).

### Limb function and joint stability

According to evaluation criteria of ISOLS (Figure 2i,j), there were five cases of excellent, four cases of good, one case of fair, no cases of poor limb function and joint stability. The rate of excellent and good was 90.0% (Table 1).

**Table 1:** Functional evaluation after operation

**Case**	**Pain**	**Function**	**Psychological endurance**	**Aided appliance**	**Walking ability**	**Gait**	**Score**
1	5	4	3	4	4	4	24*
2	4	5	4	5	5	5	28*
3	3	4	3	4	4	3	21^∆^
4	4	4	4	4	4	4	24*
5	4	2	2	3	3	2	16^#^
6	2	3	3	4	3	3	18^∆^
7	4	4	2	3	3	4	20^∆^
8	4	4	3	4	4	5	24*
9	3	3	4	4	3	2	19^∆^
10	4	5	4	4	3	4	24*

*excellent of evaluation criteria of ISOLS; ∆good of evaluation criteria of ISOLS; ^#^fair of evaluation criteria of ISOLS.

### Limb length

During the last follow-up, six patients were 1 to approximately 2 cm shorter in the operated limb than the contralateral lower limb, and four patients were 2 to approximately 5 cm shorter in the operated limb than the contralateral lower limb. There was no statistical significance of the length of the bilateral lower limb at the last follow-up (Table 2).

**Table 2:** Length of bilateral limbs during last reexamination (cm)

**Group**	**Cases**	**Limb length (cm)**
Normal limb	10	68.35 ± 3.54
Operated limb	10	65.62 ± 3.67
T		1.6815
*P*		0.0550

## Discussion

The distal femur is the main growth center of the lower limb [11] and the epiphysis of it is an important factor in the limb length of children. Assisted by effective neoadjuvant chemotherapy before operation, the effect of limb salvage surgery for malignant bone tumor had been obviously improved [12,13]. Although the tumorous bone can be resected completely by osteotomy of the unclosed epiphysis in patients who are children, the injury to the epiphysis inevitably has a negative effect on the limb growth [3]. This research attempted to preserve the epiphysis of the distal femur that was distracted by external fixation so as to preserve the knee joint function and length of the lower limb.

The tensile capacity of the epiphyseal plate is determined by collagen fibers which become thin by being squeezed by chondrocytes during development, so this area is the weakest part of the epiphyseal plate. The location of the puncture sites is an important factor for the result of the epiphyseal distraction because there is a negative effect on epiphyseal growth when the Steinman pins are punctured through the germinal layer but no damage to epiphyseal growth when the Steinman pins are punctured through the mastocyte layer [14]. The principle of epiphyseal distraction is to separate the epiphysis from the metaphysis by force on the mastocyte layer. Distraction by an external fixator can effect a complete separation from the metaphysis and avoid injury to its germinal layer by force from shear or twist [15].

The first purpose of tumor surgery is to obtain a complete tumor resection, but complications become inevitable when the resection is conservative to preserve the epiphysis, so the indications for epiphyseal distraction must be properly selected. Cañadell [9] considered the indications for epiphyseal distraction should include the following: 1) the tumor should be situated in the metaphyseal region; 2) the physeal cartilage should be open; and 3) the tumor should not transgress the physis and should be confirmed by radiography, arteriography, CT or MRI preoperatively and histological examination intraoperatively. At present, MRI is considered to be superior to X-ray and CT in determining the invasive range of malignant tumors of the limbs [16,17].

According to the image staging method of San-Julian [5], the invasion of sarcoma in the metaphysis of children is divided into three types: type I, the distance between the lesion and the epiphyseal plate is more than 2 cm; type II, the distance between the lesion and the epiphyseal plate is less than 2 cm or they are adjacent to each other; and type III, the lesion has partly invaded into the epiphysis. Although the growth plate of the cartilage can prevent the tumor from diffusing, the barriers of it are by no means impassable. Most experts consider a safe surgical tumor margin to be 5 cm outside the lesion [9], but other experts consider the epiphysis can be preserved when the distance between it and the lesion is less than 1 cm but not invaded by the lesion [18]. To guarantee a safe margin in this research, type I San-Julian staging was treated as an absolute indication for epiphyseal distraction and type II San-Julian staging was treated as a relative indication for epiphyseal distraction, but type III was a contraindication for epiphyseal distraction. According to this principle, there was no local recurrence of tumor after the surgery, indicating that the technique is safe when based on the strict indication.

At present, there are many limb salvage methods that preserve the epiphysis in malignant bone tumor, such as epiphyseal osteotomy and reconstruction using allogenic bone [19] or devitalized autologous tumor bone [20], autologous epiphysis transplantation [21]. Owing to excessive complications and limited sources, the application of autologous epiphysis is limited. Although epiphyseal osteotomy results in a relatively satisfactory outcome [20], the germinal layer of the epiphysis is damaged by osteotomy because the section of osteotomy is complanate but the interface between the epiphysis and metaphysis is sawtoothed. As a result, the growth of the epiphysis and the function of the limbs is impaired by epiphyseal osteotomy [21]. When reconstructed with devitalized autologous tumor bone, the breaking of the necrotic bone shell caused by the screws increases the possibility of tumor recurrence, so more observation for the safety and effect of this technique is needed.

The technology of epiphiseal distraction during bone lengthening has been proven to be effective and Cañadell [9] has applied a unilateral external fixator to preserve the epiphysis of tumorous bone. In order to avoid the uneven stress caused by using a unilateral external fixator, we used a bilateral external fixator to balance the force to separate the epiphysis from the metaphysis completely and effectively. After being reconstructed with allogenic bone and fixed by locking intramedullary nails, the stability of the epiphysis and the continuity of bone growth were maintained. At six months after operation, X-ray showed the gaps at both ends of the allogenic bone were healing completely. At two years after operation, X-ray showed the gap between the epiphysis and allogenic bone was well healed after the lag-screw was removed. This means that the blood supply and growth ability of the distal femur had been well preserved. At the same time, the attachment point of the cruciate ligaments and collateral ligaments at the distal femur were preserved, so that complications, such as rupture or contraction of ligaments after reconstruction, can be avoided and better knee joint function can be maintained.

Research had shown that there was partial growth inhibition in the epiphyseal growth plate after injury [22], accompanied by the closure of the epiphyseal plate and the formation of a bone bridge which seriously influence limb growth [23]. There were varying degrees of limb shortening in all patients in this study, showing that the growth of the epiphyseal plate was affected by the injury caused by distraction. There were six patients who were 1 to approximately 2 cm shorter in the operated limb than in the contralateral lower limb but this had no appreciable effect on their gait or spine growth. There were four patients under the age of 10, however, who were 2 to approximately 5 cm shorter in the operated limb than in the contralateral lower limb. This probably owing to the younger age of patients and more expected length of the operated limbs to reach the normal length, so the slight damage of the epiphyseal plate probably caused a large limb shortening of the operated limbs. Cryopreserved allograft bone has a favorable histocompatibility and bone inducing activity, weak antigenicity and strong osteogenic potential which make it effective in bone defect reconstruction after tumor resection [21]; it incorporates with host bone easily. At the same time, the epiphysis and massive allograft bone that is fixed by the screws provide a better stability for bone healing [19]. In this research, X-ray showed no loosening after the epiphysis was fixed with a massive allograft bone by lag-screw vertically. Owing to the repairing ability of children’s cartilage [24], there was no adverse effect on joint function after the cartilage flap restored.

The complications of epiphyseal distraction include infection, internal fixation loosening, nonunion or delayed union of bone, rejection and nerve injury [9,25]. Only one patient in this research suffered from poor healing of the incision, local infection and exudation from the wound because of allograft bone rejection, but the condition improved after anti-inflammation treatment. This indicates that the technology of epiphyseal distraction is safe.

## Conclusions

In this research, we preserved the epiphysis that had not been invaded by tumor through distraction by an external fixator, so that not only the integrity of the joint capsule and ligament but also the growth function of the limb was preserved. At the same time, this technique avoided not only damage to the epiphysis by osteotomy but also postoperative complications, such as contraction or rupture of ligaments and ankylosis of the knee joint. The technology of distal femur epiphysis preservation using epiphyseal distraction by an external fixator in childhood osteosarcoma may be a suitable method for limb salvage with better joint function. However, indications for this technology must include an open and uninvolved epiphyseal plate and effective neoadjuvant chemotherapy before the operation. Be restricted to the limitations such as less cases, shorter period of follow-up and evaluation for limb, further more observation were demand for the effect of the technology.

## Competing interests

The authors declare that they have no competing interests.

## Authors’ contributions

SG, Performed the conception and design of the study; Drafting the article and revising it critically for important intellectual content; Final approval of the version to be submitted. YZ, Performed the analysis and interpretation of data; Drafting the article; Final approval of the version to be submitted. QC, Performed the conception and design of the study; Revising the article critically for important intellectual content; Final approval of the version to be submitted. WY, Performed the acquisition of data; Drafting the article; Final approval of the version to be submitted. JW, Performed the acquisition of data; Drafting the article; Final approval of the version to be submitted. All authors read and approved the final manuscript.
